# Myths and Misconceptions Around Lung Cancer Causation in Palestine: Is It Time to Intervene?

**DOI:** 10.1200/GO.23.00184

**Published:** 2023-11-02

**Authors:** Mohamedraed Elshami, Ahmad Mansour, Mohammed Alser, Ibrahim Al-Slaibi, Hanan Abukmail, Hanan Shurrab, Shahd Qassem, Faten Darwish Usrof, Malik Alruzayqat, Wafa Aqel, Roba Nairoukh, Rahaf Kittaneh, Nawras Sawafta, Yousef Mahmoud Nimer Habes, Obaida Ghanim, Wesam Almajd Aabed, Ola Omar, Motaz Daraghma, Jumana Aljbour, Razan E.M. Elian, Areen Zuhour, Haneen Habes, Mohammed Al-Dadah, Bettina Bottcher, Nasser Abu-El-Noor

**Affiliations:** ^1^ Division of Surgical Oncology, Department of Surgery, University Hospitals Cleveland Medical Center, Cleveland, OH; ^2^ Ministry of Health, Gaza, Palestine; ^3^ Faculty of Medicine, Al-Quds University, Jerusalem, Palestine; ^4^ Ministry of Health, West Bank, Ramallah, Palestine; ^5^ Faculty of Medicine, Islamic University of Gaza, Gaza, Palestine; ^6^ The United Nations Relief and Works Agency for Palestine Refugees in the Near East (UNRWA), Gaza, Palestine; ^7^ Almakassed Hospital, Jerusalem, Palestine; ^8^ International Medical Corps, Gaza, Palestine; ^9^ Harvard Medical School, Boston, MA; ^10^ Department of Public Health and Primary Care, University of Cambridge, Cambridge, United Kingdom; ^11^ Faculty of Pharmacy, Al-Azhar University of Gaza, Gaza, Palestine; ^12^ Department of a Medical Laboratory Sciences, Faculty of Health Sciences, Islamic University of Gaza, Gaza City, Palestine; ^1^ ^3^Faculty of Dentistry, Al-Quds University, Jerusalem, Palestine; ^1^ ^4^Faculty of Nursing, An Najah National University, Nablus, Palestine; ^1^ ^5^Faculty of Dentistry, Al Azhar University of Gaza, Gaza, Palestine; ^1^ ^6^Faculty of Medicine, Al Najah National University, Nablus, Palestine; ^1^ ^7^Faculty of Nursing, Islamic University of Gaza, Gaza, Palestine

## Abstract

**PURPOSE:**

Having an accurate knowledge of what truly increases the likelihood of developing lung cancer (LC) may help people make better decisions about lifestyle changes that could potentially lower their risk. This study assessed current beliefs in LC causation myths among Palestinians and explored factors associated with displaying good recognition of such myths.

**METHODS:**

A national cross-sectional study was conducted from July 2019 to March 2020. A modified version of the Cancer Awareness Measure-Mythical Causes Scale was used for data collection. The awareness level of LC causation myths was determined based on the number of myths recognized to be incorrect: poor (0-4), fair (5-9), and good (10-13).

**RESULTS:**

A total of 4,817 participants completed the questionnaire of 5,174 approached (response rate = 93.1%). In total, 4,762 participants were included in the final analysis. Myths unrelated to food were more commonly recognized than food-related myths. The food-related myth most frequently recognized was eating burnt food (n = 1,427; 30.0%) followed by drinking from plastic bottles (n = 1,389; 29.2%). The food-related myth least commonly recognized was eating food containing additives (n = 737; 15.5%). The most frequently recognized myth unrelated to food was having a physical trauma (n = 2,903; 61%), whereas the least was using cleaning products (n = 1,140; 23.9%). Only 287 participants (6%) displayed good awareness. Having a chronic disease and knowing someone with cancer were associated with a decrease in the likelihood of displaying good awareness. Conversely, participants who were smoking cigarettes/shisha and those recruited from hospitals had an associated increase in the likelihood of displaying good awareness.

**CONCLUSION:**

This study found very poor awareness of LC causation myths, with only 6% recognizing ≥10 myths. Initiatives addressing LC mythical causes are needed.

## INTRODUCTION

The predicted number of newly diagnosed lung cancer (LC) cases in the Middle East and North Africa in 2018 was 79,887, with a 5-year relative survival rate of 8%.^1^ In Palestine, LC ranked third after breast and colorectal cancers. According to the Palestinian Ministry of Health statistics, LC is responsible for 7.4% and 7.8% of the total reported cancer cases in the Gaza Strip and the West Bank and Jerusalem (WBJ), respectively, with an overall incidence rate of 9.3 per 100,000 among the Palestinian population.^2^ Moreover, LC was the leading cause of cancer-related deaths, accounting for 16.6% of those reported in 2021.^2^

**CONTEXT**

**Key Objective**
Lung cancer (LC) is the leading cause of cancer-related deaths in Palestine. Therefore, this national study aimed to explore the current beliefs in LC causation myths among the Palestinian population, and examine the factors associated with good awareness of LC causation myths.
**Knowledge Generated**
The awareness of LC causation myths was poor, with only 6% of participants displaying good awareness. Factors associated with good awareness of LC causation myths included having a chronic disease and knowing someone with cancer.
**Relevance**
Health education on the myths surrounding the causes of LC may help people identify and avoid proven risk factors. Future initiatives to increase LC knowledge should target individuals with various levels of education and be designed to be simple enough for those with low literacy skills to understand.

The chance of developing LC is greatly affected by several factors, including lifestyle, environmental, and familial factors.^3,4^ Some risk factors, such as inherited mutations, cannot be modified. However, a number of modifiable lifestyle and environmental factors can be addressed, which may dramatically reduce the risk to develop cancer.^4^ For example, LC is often related to smoking, which can be stopped, lowering the risk to develop the disease.^5,6^

Accurate public knowledge of LC risk factors is a crucial element in making healthy lifestyle choices to reduce the risk of developing the disease. However, changes in behavior may lower the risk of LC only if they were focused on actual modifiable risk factors rather than myths around LC causation.^7^ Believing in LC mythical causes, such as using mobile phones, living near power lines, and feeling stressed, may be linked to concerns about developing the disease.^8^ Thus, improving people's awareness about LC myths might reduce worry about having the disease and increase people's confidence to talk about any existing symptoms that may or may not be due to LC.

Therefore, this national study aimed to (1) explore the current beliefs in LC causation myths among the Palestinian population, (2) investigate whether there is a discrepancy in the current beliefs of LC causation myths between the two main Palestinian regions, the WBJ and the Gaza Strip, and (3) examine the factors associated with good awareness of LC causation myths.

## METHODS

### Study Design and Population

The data for this national, cross-sectional study were collected between July 2019 and March 2020. The target population was Palestinian adults (age 18 years and older). Exclusion criteria were working or studying in a health-related field, having a nationality other than Palestinian, attending oncology clinics or departments during data collection, and being unable to complete the questionnaire.

### Ethics Approval

On June 24, 2017, the Helsinki Committee in the Gaza Strip and the Human Resources Development Department in the Palestinian Ministry of Health granted their ethical approval before data collection. Additionally, on June 26, 2017, the Islamic University of Gaza's Research Ethics Committee granted their ethical approval as well. The study's objectives were thoroughly explained to the participants, with a focus on the fact that their participation was entirely voluntary. Before beginning the questionnaire, study participants provided written informed consent, and data were collected anonymously. All the methods of the study were carried out in accordance with relevant local guidelines and regulations.

### Sampling Methods

Using convenience sampling, eligible participants were visitors to governmental hospitals, primary health care centers, or public spaces in 11 of 16 Palestinian governorates. Public spaces included marketplaces, mosques, churches, restaurants, parks, transit hubs, and others. Recruiting participants from various geographic locations was intended to increase the representativeness of the study cohort.^9-13^

### Questionnaire and Data Collection

Data were collected using a modified version of the Cancer Awareness Measure-Mythical Causes Scale (CAM-MYCS). The CAM-MYCS is a reliable tool for exploring public perceptions of cancer myths.^14^ Two bilingual health care professionals initially translated the original CAM-MYCS into Arabic, and then two further bilingual health care professionals translated it back into English. Five specialists in thoracic oncology, public health, and survey design evaluated the content validity and translation accuracy of the Arabic version. A pilot study (n = 68) was conducted to evaluate the clarity of the questions in the Arabic version of the questionnaire. The final analysis did not include participants from the pilot study. Internal consistency was evaluated using Cronbach's alpha, and a satisfactory result of 0.78 was obtained.

The questionnaire comprised two sections. The sociodemographic characteristics of the study participants were described in the first section. In the second section, participants were asked to identify 13 myths around LC causation. Twelve of the 13 examined myths around LC causation were adapted from the original CAM-MYCS.^14^ Eating burnt food was added to the questionnaire as it was deemed important to assess its frequency as a health misbelief among Palestinians.^15^

For the purposes of this study, the original CAM-MYCS was modified. To reduce the possibility of participants answering questions at random, the original CAM-MYCS questions with correct/incorrect/unsure responses were changed into five-point Likert scale questions (1 = strongly disagree, 2 = disagree, 3 = not sure, 4 = agree, 5 = strongly agree). The participants' answers were then categorized into correct and incorrect categories, where responses with strongly disagree or disagree were deemed correct and all other responses were deemed incorrect.

The electronic tool Kobo Toolbox (Kobo, Cambridge, MA) was used in a face-to-face interview with the participant for data collection. This safe tool can be used both offline and online on mobile devices.^16^ All data collectors had a medical background and received training on the use of Kobo Toolbox, obtaining informed consent, recruiting participants, and facilitating the completion of the questionnaire.

### Statistical Analysis

Starting at age 45 years, the percentage of new LC cases rises significantly.^17^ Consequently, using this cutoff point, participants' ages were categorized into two categories: 18-44 and 45 years. Since the minimum wage in Palestine is 1,450 New Israeli Shekel (NIS; about $450 in US dollars), monthly income was also categorized into two categories: <1,450 NIS and ≥1,450 NIS.^18^

Descriptive statistics were used to describe participant characteristics. Categorical variables were described using frequencies and percentages, whereas continuous variables, which were non-normally distributed, were described using median and IQR. The Kruskal-Wallis test was used to compare baseline characteristics of participants recruited from the Gaza Strip and those recruited from the WBJ if the characteristic was continuous, while Pearson's chi-square test was used if the characteristic was categorical.

Myths around LC causation were categorized into two categories: food-related and food-unrelated. Recognition of each LC mythical cause was described using frequencies and percentages with comparisons between participants from the Gaza Strip and those from the WBJ performed by Pearson's chi-Square test. This was followed by running multivariable logistic regression analyses to examine the association between participant characteristics and recognizing each LC myth as incorrect. The multivariable analysis adjusted for age group, sex, educational level, monthly income, occupation, place of residence, marital status, having a chronic disease, knowing someone with cancer, smoking history, and site of data collection. This model was determined a priori based on previous studies.^7,14,19-22^

A scoring system was used to assess the level of awareness of myths around LC causation. Similar scoring systems were also used in previous studies.^9-13^ One point was given for each correctly recognized LC myth. Awareness level was determined based on the total score (ranging from 0 to 13), which was calculated and categorized into three categories on the basis of the number of myths identified as incorrect: poor (0-4), fair (5-9), and good (10-13). Recognition of LC myths by participants from the Gaza Strip and those from the WBJ was compared using Pearson's chi-square test. A multivariable logistic regression analysis was used to examine the association between participant characteristics and displaying good recognition of myths around LC causation as incorrect. The same aforementioned multivariable model was used.

Missing data were hypothesized to have occurred completely at random and thus, complete case analysis was used to handle them. Data were analyzed using Stata software version 17.0 (StataCorp, College Station, TX).

## RESULTS

### Participant Characteristics

A total of 4,817 participants agreed and completed the questionnaire of 5,174 approached (response rate = 93.1%). However, 55 participants were excluded, 31 because of missing data and 24 because of not meeting the inclusion criteria. Therefore, 4,762 participants were included in the final analysis; 2,742 were from the WBJ and 2,020 were from the Gaza Strip. Participants from the WBJ were older, had lower education levels but higher monthly income, more often smoked cigarettes or shisha, and suffered from more frequent chronic diseases than participants from the Gaza Strip (Table 1).

**TABLE 1:**
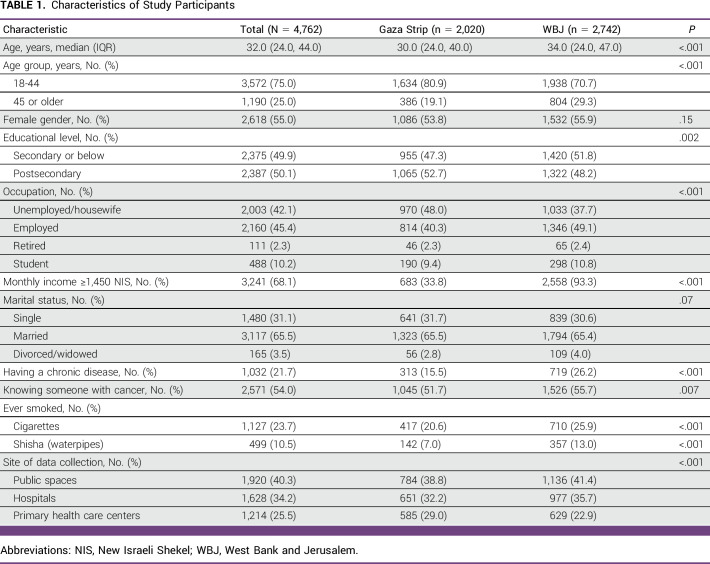
Characteristics of Study Participants

### Recognition of LC Causation Myths

Overall, myths unrelated to food were more commonly recognized to be incorrect than food-related myths. The food-related myth most frequently recognized to be incorrect was eating burnt food (n = 1,427; 30%) followed by drinking from plastic bottles (n = 1,389; 29.2%; Table 2). The food-related myth least commonly recognized to be incorrect was eating food containing additives (n = 737; 15.5%). The most frequently recognized myth unrelated to food was having a physical trauma (n = 2,903; 61%), whereas the least was using cleaning products (n = 1,140; 23.9%; Table 2).

**TABLE 2:**
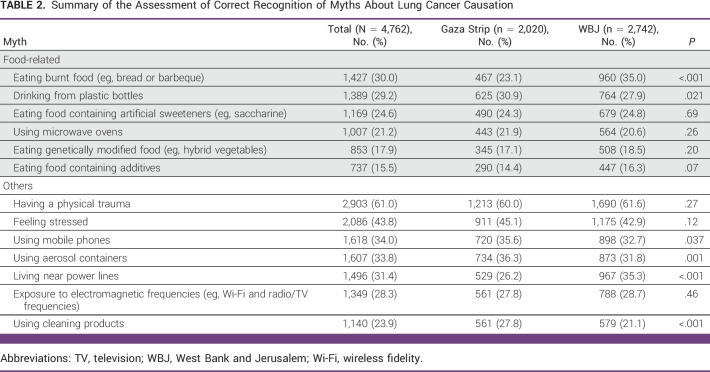
Summary of the Assessment of Correct Recognition of Myths About Lung Cancer Causation

### Good Recognition of LC Causation Myths and Its Associated Factors

Only 287 participants (6%) displayed good awareness (ie, correct recognition of 10 or more of 13 LC causation myths), while the greatest number of participants (n = 2,907; 61.1%) had poor awareness (ie, correct recognition of less than 5 of 13 LC causation myths; Table 3). Participants from both the WBJ and the Gaza Strip had a similar likelihood to have good awareness (6.4% *v* 5.5%). On multivariable analysis, having a chronic disease and knowing someone with cancer were associated with a decrease in the likelihood of displaying good awareness level (Table 4). Conversely, participants who were smoking cigarettes/shisha and those who were recruited from hospitals had an increase in the likelihood of displaying good awareness.

**TABLE 3:**
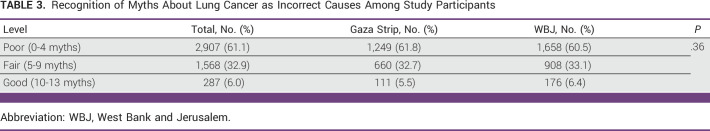
Recognition of Myths About Lung Cancer as Incorrect Causes Among Study Participants

**TABLE 4:**
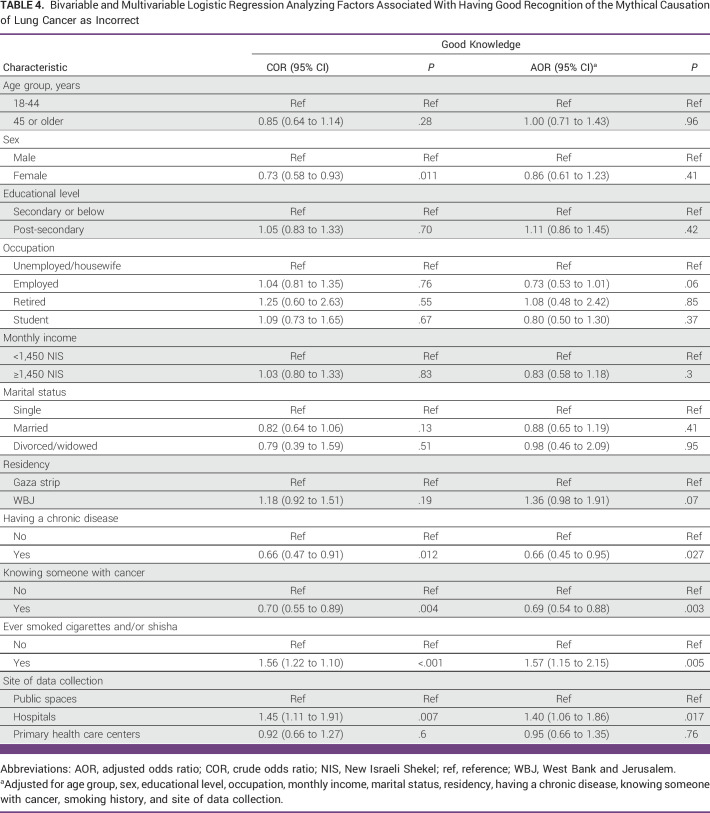
Bivariable and Multivariable Logistic Regression Analyzing Factors Associated With Having Good Recognition of the Mythical Causation of Lung Cancer as Incorrect

### Association Between Participant Characteristics and Recognition of Food-Related Myths Around LC Causation to Be Incorrect

Participants who knew someone with cancer were less likely than participants who did not to recognize all LC food-related mythical causes except eating food containing artificial sweeteners, where no associated difference was found (Table 5). By contrast, participants recruited from hospitals were more likely than those recruited from public spaces to recognize all LC food-related myths as incorrect, except eating food containing artificial sweeteners and eating genetically modified food, where no associated differences were observed. Additionally, participants from the WBJ were more likely than participants from the Gaza Strip to recognize half of the LC food-related myths to be incorrect, namely eating burnt food (odds ratio [OR], 2.01 [95% CI, 1.68 to 2.40]), eating genetically modified food (OR, 1.45 [95% CI, 1.17 to 1.78]), and eating food containing additives (OR, 1.53 [95% CI, 1.22 to 1.91]). Both groups of participants who completed or did not complete higher education (ie, postsecondary school education) had a similar likelihood to recognize all LC food-related myths to be incorrect.

**TABLE 5:**
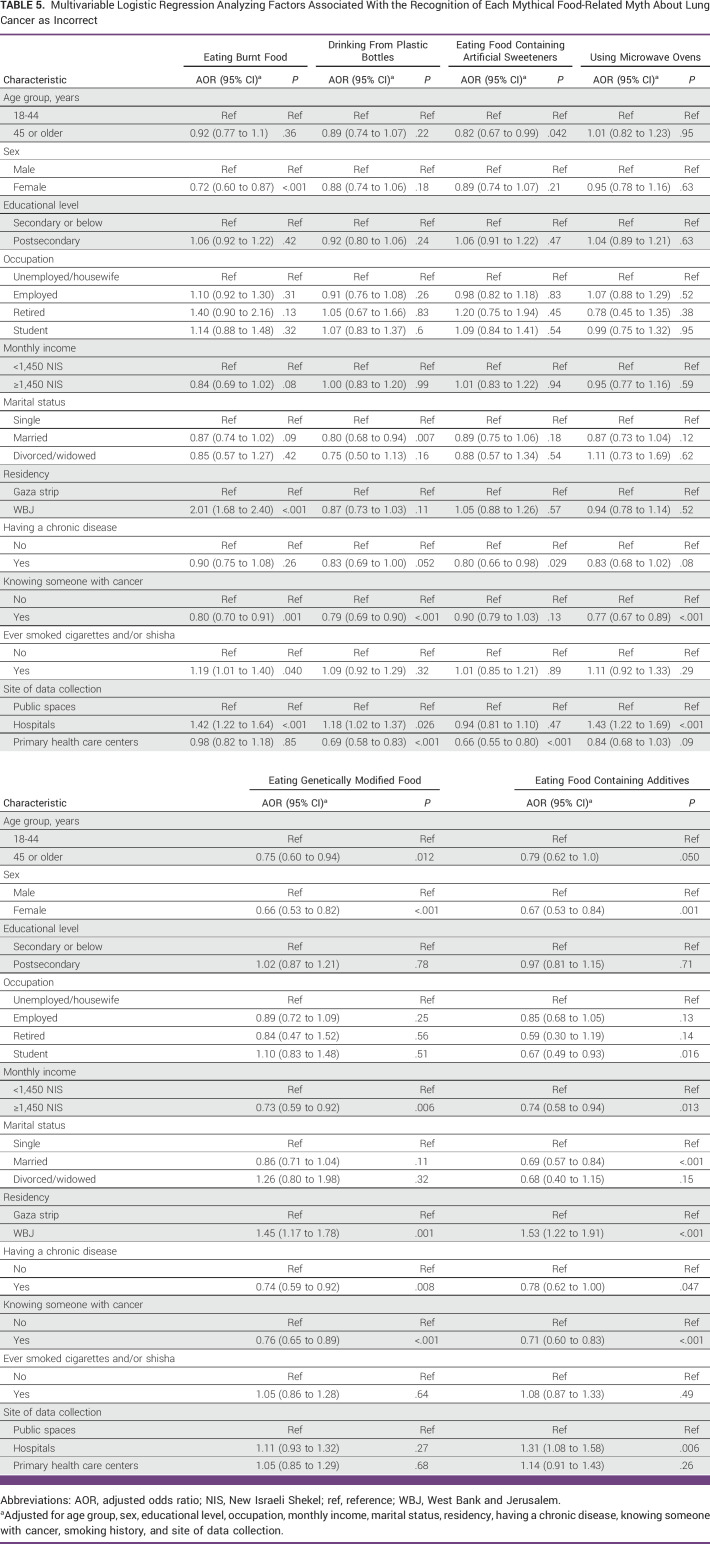
Multivariable Logistic Regression Analyzing Factors Associated With the Recognition of Each Mythical Food-Related Myth About Lung Cancer as Incorrect

### Association Between Participant Characteristics and Recognition of Food-Unrelated Myths Around LC Causation to Be Incorrect

Female participants were less likely than male participants to recognize all other myths to be incorrect, except using aerosol containers and using cleaning products, where no associated differences were observed (Table 6). Participants who knew someone diagnosed with cancer were less likely than participants who did not to recognize five of the seven LC food-unrelated myths to be incorrect. Participants with higher levels of education were more likely to recognize having a physical trauma (OR, 1.24 [95% CI, 1.08 to 1.41]), less likely to recognize exposure to electromagnetic frequencies (OR, 0.81 [95% CI, 0.70 to 0.93]), and had similar likelihoods to recognize all other LC food-unrelated myths as incorrect compared with participants with lower levels of education.

**TABLE 6:**
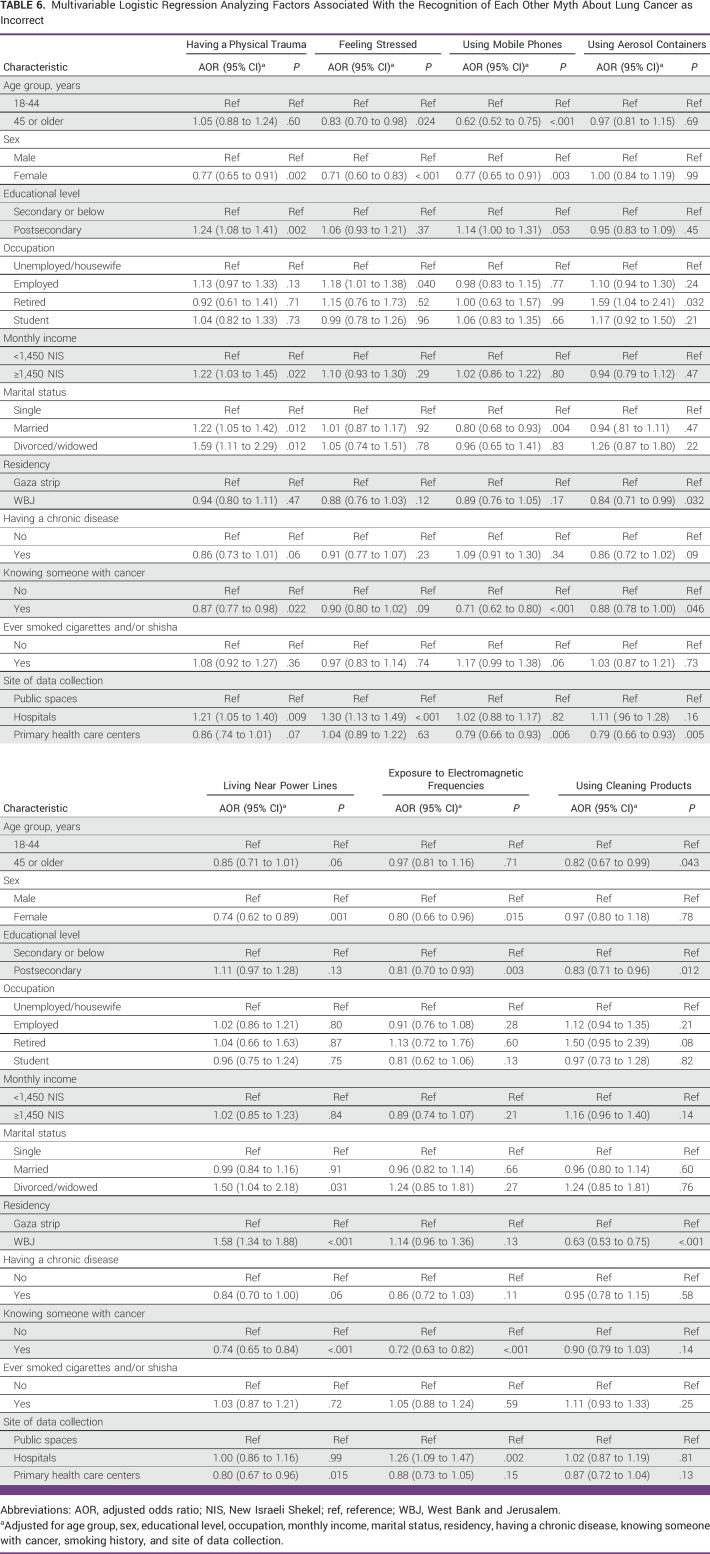
Multivariable Logistic Regression Analyzing Factors Associated With the Recognition of Each Other Myth About Lung Cancer as Incorrect

## DISCUSSION

In this study, good recognition of the myths around LC causation was demonstrated by only 6% of study participants, where interpreting ≥10 myth as incorrect was the recommended state of knowledge. Participants from the Gaza Strip and the WBJ were equally likely to display good awareness of the examined myths. Hospital visits and smoking cigarettes or shisha were associated with a greater likelihood of showing good recognition of the examined myths to be incorrect. Overall, myths unrelated to food were more frequently acknowledged as incorrect by participants than those linked to food.

Reductions in cancer risk can be achieved by targeting various environmental and lifestyle risk factors that are modifiable, such as low fruit and vegetable intake, high consumption of red and processed meat, low physical activity, smoking, and alcohol consumption.^23^ Health beliefs in cancer myths to be possible causes for cancer may be linked to increased worry about developing the disease.^8^ In addition, depressive symptoms were associated with cancer stigma and self-blame.^24^ In particular, LC has greater stigma scores than other cancers, probably because of its link with smoking,^25^ which might delay seeking medical help.^26^ Thus, greater recognition of cancer myths to be incorrect might reduce cancer worry, boost people's confidence, and encourage early presentation, which in turn might improve prognosis.

Consequently, to decrease LC risk, people's lifestyle choices should be targeted to avoid actual and modifiable risk factors and avoid distraction by addressing myths of LC causation that are not evidence-based.^7^ Assessing the public knowledge of actual and mythical LC causes may aid in directing actions aimed at raising awareness.^14^ A previous national study in Palestine showed that only 50% displayed good awareness of evidence-based LC risk factors.^11^ This study adds to the literature by providing data on the awareness of LC mythical causes in the Palestinian community, where disagreeing on the questionnaire items (ie, LC causation myths) was the recommended state of knowledge.

LC myths unrelated to food were more frequently recognized than those related to food. Redeker et al^27^ surveyed 4,233 participants in the United Kingdom and found that 34% thought of stress as a risk factor for cancer. Similarly, Shahab et al^7^ showed that stress was, beside food-related myths, the most frequently recognized LC causation myth. However, stress was the second most commonly recognized myth to be incorrect in our study, while the most common misbelief recognized was having a physical trauma. Munir et al^28^ found in their study from Pakistan that the myth cancer is contagious was the most widely identified myth, followed by aerosol and powerlines. However, fertilizer/pesticide spray, microwave ovens, and drinking from plastic bottles were the least recognized myths. Although there is a degree of overlap between commonly recognized and unrecognized myths, there appear to be differences among various settings. Therefore, data about such details can be useful in the design and implementation of targeted educational campaigns to improve cancer awareness.

Shahab et al^7^ reported that better recognition of cancer myths to be incorrect was more frequent among younger participants. However, in concordance with this study, the study by Munir et al^28^ did not find a significant association between participants' age and their recognition of the examined myths. In contrast to this study, the study from Pakistan found that participants who knew someone with cancer were not more able to recognize cancer myths as incorrect. Interestingly, having a good understanding of LC myths was not associated with being recruited from primary health care centers. All these findings contradict with a previous national study from Palestine that showed that older age, knowing someone with cancer, and visiting primary health care centers were all associated with higher recognition of actual LC risk factors.^11^ A plausible explanation for this could be the absence of appropriate information about LC causation myths provided to the public, especially those attending governmental primary health care centers. Thus, future educational interventions should be tailored to address the needs of these groups. Short training sessions were found to significantly increase people's recognition of cancer myths and their ability to identify them to be incorrect.

Higher levels of education were linked to better awareness of the risk factors for lung, colorectal cancer, breast, and ovarian cancers.^10-13^ However, in our study as well as two other studies, it was not associated with better recognition of cancer myths to be incorrect.^7,28^ This means that campaigns should target people of all levels of education to achieve better awareness; however, the way of presenting such information should be tailored depending on educational and cultural background.^7^

The provision of health information on LC causation myths might further improve correct recognition of LC risk factors versus mythical causes. It might be possible to implement brief training sessions in Palestine that were shown to greatly raise people's knowledge of cancer myths.^28^ Interventions to raise awareness should target people with different educational levels and tailored to be easily understood by people with poor literacy. Furthermore, it is necessary to give more emphasis on positive adoption of proven protective factors besides focusing on education against adoption of LC causation myths.^7^

The results of this study should be interpreted while considering some limitations. The generalizability of the results might be limited because of the use of convenience sampling. Nonetheless, the large number of participants, the variety of the included geographic areas, and the high response rate may have mitigated this limitation. The fact that participants with medical backgrounds and visitors to cancer departments were all excluded may have reduced the number of participants with a presumably better knowledge. However, this was done in an effort to increase the relevance of measured awareness to the general public.

In conclusion, very small proportion of study participants (6%) exhibited good awareness of the examined myths around LC causation to be incorrect. Hospital visitors and cigarette or shisha smokers had higher likelihood of displaying good recognition of LC causation myths. Participants recognized LC myths unrelated to food more frequently than those related to food. Future educational interventions should include guidance on behavior modifications that target known risk factors rather than myths that are incorrectly linked to LC causation.

## Data Availability

The data set used and analyzed during the current study is available from the corresponding author on reasonable request.
